# First-In-Human Phase 1 Clinical Trials – A Single-Center Experience In The Era Of Modern Oncotherapeutics

**DOI:** 10.1038/s41598-020-64906-4

**Published:** 2020-05-13

**Authors:** Ravi K. Paluri, Peng Li, Ashley Anderson, Lakshminarayana Nandagopal, Traci McArdle, Matthew Young, Franscisco Robert, Gurudatta Naik, Mansoor Saleh

**Affiliations:** The University Of Alabama at Birmingham, O’Neal Comprehensive Cancer Center, Department of Medicine, Division of Hematology Oncology, Birmingham, US

**Keywords:** Cancer, Cancer therapy, Drug development

## Abstract

In the era of precision medicine the treatment options for cancer patients and subsequent outcomes are expected to improve. We present a review of patients enrolled in first-in-human Phase1 trials at University of Alabama at Birmingham. Between 1/2015–6/2017, 162 cancer patients (whole cohort, WC) were enrolled on phase1 studies receiving either targeted therapy (TT) or immuno-therapy (IOT). We assessed 90 day mortality (90DM) and time to treatment failure (TTF) to determine the predictors. Of the WC (122 (TT), 40 (IOT)), 90 (56%) received ≥ 2 prior therapies and 38 (24%) ⩾ 5 prior therapies. Overall, Grade 3 or 4 events were observed in 33% (WC) vs 31% (TT) vs 38% (IOT). The 90DM was 9.3% (WC) vs 7.4% (TT) vs 15% (IOT). The median TTF was 4.2 months vs 4.5 m vs 3.6 m. The number of lines of prior therapy and performance status were identified as outcome predictors. Our data reflects the new trend in precision oncology where majority received non-cytotoxic therapeutic interventions. The observation that number of lines of prior therapy and performance status predictive of PFS and 90DM emphasizes the need to consider phase1 trials earlier, preferably upon progression following definitive therapy.

## Introduction

Experimental therapeutics programs have been in place at major academic centers for over four decades. The emergence of molecular targeting agents and the recent introduction of immuno-oncology drugs have expanded the scope and eligibility for first-in-human trials. Improved understanding of tumor biology coupled with the ability to screen for tumor associated targets, as well as, genetic alterations have heralded the era of personalized (personalized or precision) cancer treatment. Molecular targeting agents with their improved tolerability and sustained responses compared to conventional cytotoxic chemotherapy have contributed to remarkable improvements in clinical outcomes. Dramatic phase 1 observations of anti-tumor activity of novel molecules in the relapsed or refractory setting have often led to their investigation as monotherapy or in combinatorial strategies early in the course of cancer treatment. Phase 1 clinical trials have thus evolved from the traditional role of dose and toxicity-finding studies to innovative enrichment study designs which match patients with study agents, thus increasing the potential of clinical efficacy, even in the early dose escalation setting.

While chemotherapeutic agents still have an important role in oncology, the era of precision medicine is beginning to revolutionize treatment options and outcomes for cancer patients. The UABOCCC, Phase 1 Clinical Trials Program was formally established in 2015, in an effort to offer novel first-in- human therapeutic clinical trials to cancer patients in a one-stop-shop setting. The program was initiated with 6 clinical trials and rose to 17 clinical trials by 2017. We enrolled 60 patients in our first year and close to 100 by 2017. This single-center, retrospective analysis was performed to assess clinical outcomes and the predictors of survival and efficacy in patients during the first two and a half years of our program. Our program was unique in that all patients received targeted or immuno-oncology agents as the backbone of their treatment^1,2^. Previous published work ^3,4^ included patients who had received cytotoxic chemotherapeutic drugs. There remains an unmet need to identify predictors of outcome and survival in patients treated exclusively on targeted or immuno-oncology Phase 1 studies.

## Materials and Methods

Our analysis included all adult oncology patients that enrolled and completed at least one cycle of treatment on a phase 1 oncology first-in-human clinical trial conducted at UABOCCC. We included patients treated both on hematologic and solid tumor trials. One hundred and sixty two patients were enrolled from January 2015 through June 2017, and formed the basis for our retrospective study. We collected data on age, gender, race, height, weight, date of diagnosis, number of prior treatment regimens, study agent mechanism of action, previous genetic testing history and results, cycle 1 day 1 date, best radiographic response and date, date of treatment discontinuation and reason for discontinuation, date of last follow up, mortality, baseline and end of treatment performance status and laboratory values on all patients. We also captured all Grade 3 and 4 adverse events, serious adverse events, and dose limiting toxicities, if applicable. Patients treated with agents targeting a pathway or cell-surface receptor (e.g. Tyrosine Kinase, Fibroblast Growth Factor receptor, MET) were included under targeted therapy (TT) cohort, whereas patients who received agents specifically designed to activate the immune effector system (e.g. Programmed death – 1 or Programmed death ligand – 1, CTLA-4, IDO inhibitors or GITR agonist) were included under immuno-oncology therapy (IOT) cohort. The primary objective is assessment of 90 day mortality (90DM) All patients included in our analysis met the respective protocol specific eligibility and all protocols were approved by Institutional Review Boards and conducted in accordance with Good Clinical Practice Guidelines. All protocols were registered on clinicaltrials.gov. This retrospective study received expedited approval by University of Alabama at Birmingham Institutional Review Board since it involved retrospective data analysis without patient identifiers. The primary objectives of this study were to evaluate clinical benefit as measured by 90-day mortality (90DM); and the secondary objective is to assess the clinical benefit in terms of time to treatment failure (TTF), overall survival (OS) and predictors of clinical outcomes in this unique Phase 1 patient population.

### Endpoints

The primary endpoint is 90 day mortality (90DM). The secondary endpoints are time to treatment failure (TTF) and overall survival. TTF for evaluable patient was defined as the time elapsed between start of protocol specific study treatment (cycle 1/day1) until end of treatment visit (EOT) due to clinical or radiological progression or disease-related death, whichever occurred first; if no evidence of progression was observed at the last follow-up, TTF was censored at the time of last radiological evaluation. OS was defined as the interval between the date of diagnosis and the death. For patients still alive during the follow up period, OS was censored at the date of last follow-up. Clinical benefit rate (%) was defined as the combination of complete response (CR), partial response (PR) and stable disease (SD) that was maintained for at least 6 months.

### Study assessments

#### Response assessment

As in all Phase I studies, baseline tumor measurements were performed within 2–4 weeks prior to commencement of treatment. Tumor measurements were repeated every 6–8 weeks in accordance with the corresponding study protocols using Response Evaluation Criteria In Solid Tumors (RECIST) version 1.1 or immune-modified response evaluation criteria (iRECIST) for protocols including immune-oncology agents.

### Safety

Safety was evaluated based on findings at baseline, at protocol defined intervals during treatment, and for 28 days after completing study therapy. Safety assessments included physical examination and toxicity assessment as well as laboratory studies including hematologic parameters, serum chemistry, and urine analysis performed as per protocol. Toxicity data were collected from electronic medical records (EMR) source document and study case report forms (CRFs). Toxicities were characterized by type, frequency, seriousness, relationship to study drug, and were graded using the National Cancer Institute Common Terminology Criteria for Adverse Events (NCI CTCAE), version 4.03. Dose limiting toxicities (DLT) were assessed during the first cycle as determined by the respective protocols.

### Statistical methods

Descriptive statistics (mean, median, standard deviation and ranges for continuous data, and percentages for categorical data) were used to summarize patient characteristics, treatment administration, safety, and efficacy. Response rates along with corresponding 95% confidence intervals were calculated, based on the exact binomial distribution. TTF and OS were evaluated with Kaplan-Meier estimates. The relationship between clinical characteristics and clinical outcomes, TTF and 90DM was assessed using univariate and multivariate logistic regression to determine the predictors of outcomes. All analysis was performed using SAS 9.4 (Cary, NC) and the p-values smaller than 0.05 will be considered significant.

### Ethical approval

This article does not contain any studies with animals performed by any of the authors. All procedures performed in studies involving human participants were in accordance with the ethical standards of the institutional and/or national research committee and with the 1964 Helsinki declaration and its later amendments or comparable ethical standards.

### Compliance with ethical standard

 This is a retrospective study that does not require valid consent from patient. Therefore ethics approval was not required. The Study was performed in accordance with the Declaration of Hesinki.

## Results

Patient Characteristics are provided on Table 1. The patients with wide spectrum of underlying malignances were treated: Hematological malignancies (leukemia, lymphoma, myeloma) at 34%, Gynecological tumors (14%), Breast (11%), head and neck (9%), Gastrointestinal (7%), lung (6%), Sarcoma (5%), melanoma (4%), genitourinary (4%) and pancreatobiliary (4%). The UAB Phase 1 Clinical Trials Program enrolled 162 patients with advanced cancers on 17 separate protocols with 40 patients (25%) receiving immuno-oncology therapy (IOT) and 122 patients (75%) received targeted therapy (TT). Patient characteristics were summarized in Table 1. Median numbers of treatments prior to enrolling on a Phase 1 trial were 3. Forty five percent of patients received prior radiation. The median age at the time of enrollment was 54 years (range 16–83). The study population included 76 men (47%) and 86 women (53%); 129 (80%) Caucasians and 32 (22%) African Americans. All patients enrolled on our first-in-human Phase 1 clinical trials were required to have an ECOG performance status of 0 or 1; one third had an excellent performance status (ECOG 0) at the time of enrollment. Next generation sequencing (NGS) was available on 49 of 162 (30%) patients. Activating mutation was identified in in 25 of these 49 patients (51%).

**Table 1:** Patient characteristics and baseline data at the time of enrollment in Phase 1 clinic.

Variable	N (%)(range)
Median Age	54.4
Caucasian	129 (80%)
African Americans	32 (20%)
Male	76 (47%)
ECOG 0	59 (38%)
Immunotherapy (IOT)	40 (25%)
Targeted treatments (TT)	122 (75%)
Base line Albumin	4 (2.8–4.8)
Base line LDH	190.5 (109.0–895.0)
Prior Rx ≤ 2	72 (44%)
Prior Rx 3–4	52 (32%)
Prior Rx ≥ 5	38 (24%)
Prior Radiation	72 (45%)
Hematological malignancies	55 (34%)
Non-Hematological Malignancies	107 (66%)

N = 162

### Adverse events

The Table 2 outlines the frequency of grade 3 or 4 adverse events (AE) observed in the immuno-oncology therapy or targeted therapy groups. Overall, the incidence of AE (all grade) between IOT and TT was similar (62% vs 60% respectively) and high grade (3 and 4) toxicity was slightly higher in IOT (38%) compared to TT (31%). We had low threshold in admitting the patients treated with IO drugs from safety perspective and this likely contributed to higher incidence in IOT group. No treatment related mortality was observed.

**Table 2:** Number of patients with Grade 3 and 4 adverse events.

	IOT	T T
Hospitalization	6	14
GI (N/V,D,Hepatic)	1	4
Thrombocytopenia	0	3
Anemia/transfusion	0	2
Pain	2	3
Infection	4	9
GI bleed	1	2
Hemoptysis	1	1

IOT: pts treated on immunotherapy clinical trials; TT: Pts treated on targeted therapy clinical trials; All Grade adverse events occurred in 62% (IOT) and 60% (TT); Grade 3/4 toxicities as outlined above occurred in 38% (IOT) and 31% (TT); N/V: Nausea/Vomiting, D: Diarrhea, Hepatic: Hepatic dysfunctions / transaminitis

### Response assessments

The clinical outcomes for whole cohort (WC), IOT and TT cohorts are shown in Table 3. At a median follow up of 17.2 months, the median overall survival (OS) was 26.6 months (WC), 19.6 months (IOT) and 33.3 months (TT) respectively. The 90day mortality (90 DM) in our cohort was 9.3% (WC) vs 15% (IOT) vs 7.4% (TT). The median follow up for TTF was 8.5 months. The time to treatment failure (TTF) was 4.2 months (WC) vs 3.7month (IOT) vs 4.5months (TT) respectively. Complete response (CR) in these pretreated patients was 9% (WC) vs 5% (IOT) vs 11% (TT). Overall, the clinical benefit rate CR + PR (partial response) + SD > 6months) was 67% (WC) vs 65% (IOT) vs 67% (TT). Stable disease (SD) for ⩾6 months was observed in 37% vs 35% vs 38% respectively. The one year survival for the patients who achieved SD ⩾6 month (n = 23) compared to those who achieved overall response (CR + PR, n = 42) was 95% vs 92% (p = 0.5).

**Table 3:** Results - efficacy estimates.

	Total (N = 162)	IOT (N = 40)	TT (N = 122)
90 day mortality	15 (9.3%)	6 (15.0%)	9 (7.4%)
TTF (months), median (95%CI)	4.2 (3.4, 6.4)	3.7 (2.3, 6.4)	4.5 (3.6, 10.4)
OS (months), median (95%CI)	26.6 (19.3, -)	19.6 (5.2, -)	33.3 (19.3, -)
Best Response			
Complete Response	15 (9.3%)	2 (5.0%)	13 (10.7%)
Partial Response	27 (16.7%)	7 (17.5%)	20 (16.4%)
Stable Disease	66 (40.7%)	17 (42.5%)	49 (40.2%)
Progressive Disease	43 (26.5%)	12 (30.0%)	31 (25.4%)
Not Assessed	11 (6.8%)	2 (5.0%)	9 (7.4%)
Stable disease (med,months)	3.8 (0.4–29.1)	3.5 (0.6–23.9)	3.9 (0.4–29.1)
0–3 months	70 (43.2%)	18 (45.0%)	52 (42.6%)
3–6 months	32 (19.8%)	8 (20.0%)	24 (19.7%)
>6 months	60 (37.0%)	14 (35.0%)	46 (37.7%)

IOT: pts treated on immunotherapy clinical trials; TT: Pts treated on targeted therapy clinical trials; TTF:Time to treatment failure; OS Overall survival

Table 4 provides the predictors of clinical outcome. Performance status and number of prior therapies appeared to be important predictors of clinical outcome in terms of 90 DM and TTF. On univariate analysis the predictors of 90DM that trended towards statistical significance for the WC were performance status (ECOG 0 vs 1, OR = 0.28, p = 0.07) and number of prior treatments (Rx ≤ 2 vs >2, OR = 0.31, p = 0.06).Significant predictors for TTF were again baseline performance status (ECOG 0 vs 1, OR = 0.65, p = 0.035) and number of prior treatments (Rx ≤ 2 vs >2, OR = 0.48, p = 0.004). Overall patients with excellent performance status, ECOG 0 and ≤2 prior treatments at the time of enrollment had low 90 DM and better TTF. Patients treated with targeted agents appear to have better TTF compared to IO drugs. The predictors for survival in a multivariate analysis for the WC were number of prior therapies (Rx ≤ 2 vs >2; HR 0.54, p = 0.02) and type of drug, in favor of TT, (HR = 0.54, p = 0.03). Patients receiving less than 2 treatments or treated on TT clinical trials had better OS. There was a trend towards significance with baseline neutrophil to lymphocyte ratio where the ratio >4 appeared to be associated with better OS compared to <4 (HR = 0.62, p = 0.07).

**Table 4:** Predictors of clinical outcomes.

OUTCOME	Predictor Odds Ratio (95%CI); p
90 DM	ECOG 0.28, (0.07–1.12); p = 0.07
	Prior Rx 0.31, (0.09–1.08); p = 0.06
TTF	ECOG 0.65, (0.43–0.97); p = 0.035
	Prior Rx 0.48, (0.32–0.72); p = 0.0004
No response	Prior Rx 0.22, (0.10–0.47); p < .0001
	TT vs IOT 0.36, (0.14–0.94); p = 0.037

90DM: 90-day mortality; TTF: time to treatment failure; IOT: pts treated on immunotherapy clinical trials; TT: Pts treated on targeted therapy clinical trials; Prior Rx: previous therapies; ECOG: Eastern Cooperative Oncology Group performance status.

Progression at first restaging was observed in 26.5% (WC) vs 30.0% (IOT) vs 25.4% (TT). The independent factors predictive of poor response or progression at first restaging were number of prior therapies and type to drug (TT vs IOT) in the clinical trial. Patients who received less than 2 systemic therapies prior to enrollment into the study responded better to the study treatment (OR 0.22, 95%CI (0.10–0.47), p < 0.0001). Patients treated with IOT drugs compared to TT drugs had lower chance of response compared to IOT drugs (OR 0.36, 95%CI (−0.14,0.94), p = 0.03).

In terms of laboratory parameters, there was no significant variation between pre and end of treatment (EOT) laboratory values for platelet count, white cell count, neutrophil, creatinine, transaminases and bilirubin. However, the median hemoglobin, lymphocyte count, serum albumin and LDH were significantly lower at EOT compared to pre-study levels.

## Discussion

In oncology phase 1 clinical trials are traditionally designed to establish the maximum tolerated dose of anticancer therapies. However, with the advent of molecularly targeted and immuno-oncology treatment, the objectives of early phase trials have trended to focus on understanding the novel mechanism of action of these antitumor agents and the tumor biology^5^. Consequently phase 1 studies frequently select patients who will likely benefit from the proposed mechanism of action of the novel agent. Most studies also require pre and post-treatment biopsies to identify biologic markers of anti-tumor activity. Critics have raised a fundamental concern about participation on phase 1 clinical trials in view of the dismal response rate of 5% ^6,7^. In recent years, however, early phase trials have reported encouraging tumor shrinkage even in the context of a dose escalation design. The data from our single center cohort of 162 patients treated with TT or IOT over the past 2.5 years supports this observation. All the patients in our cohort participated in either IOT or TT first in human dose finding / escalation trials. There were no chemotherapy based phase 1 trials during this reported period. A recent retrospective study that evaluated phase 1 trials published between 2014 and 2015, with primary end point of response rate, demonstrated an overall response rate of 19.8%. The study population included patients who received both targeted agents and conventional chemotherapy with approximately ~20% of the patients receiving cytotoxic chemotherapy. The predictor of overall response was observed to be the trials investigating a single tumor type^8^. With the focus of research shifting from cytotoxic agents to targeted drugs, our objective was to understand drug activity of newer agents in a pre-defined cohort of patients. Our results indicate that phase 1 trials that are based on an enrichment design with focus on histologic characteristics or molecular targets were associated with a higher probability of clinical benefit. Our data also demonstrated a higher probability of an objective tumor response among patients enrolled in phase 1 trials than has been historically reported. Clinical trials with major proportion of targeted therapies, were thought to have better tolerance and favorable toxicity profiles than traditional cytotoxic agents^9^. There was no treatment or toxicity related mortality observed in our cohort. No significant difference was observed in the incidence of grade 3–4 toxicities between patients treated with targeted or immunotherapies.

We identified clinical characteristics predictive of shorter TTF and 90 DM. The number of lines of prior systemic therapy and ECOG performance status was identified as predictors of TTF and 90DM for the WC, TT and IO. Various prognostic scores such as Royal Marsden Hospital model, have been previously evaluated^10^ but have diminishing relevance in the era of personalized (need to decide on personalized vs precision) treatment approaches. IOT- specific prognostic scores are also being currently studied^11^. Larger studies are needed to evaluate predictors of outcomes for the trials focused exclusive on targeted and immunotherapy.

Our study is the first to specifically report on the clinical outcomes of phase 1 trials exclusively involving TT or IOT and no cytotoxic chemotherapy. The outcomes from our study cohort appears comparable to recent data published from other institutions that employed a mixture of IOT, TT and cytotoxic chemotherapy in their early phase studies^12,13^ This comparable outcome may well reflect the impact of a targeted approach in recent phase 1 trials. A review in 2005 demonstrated response rates approaching 11% in phase 1 studies conducted at the National Cancer Institute^14^. Historically, 90 DM approached 15–20% in phase 1 trials ^15–17^. A more recent retrospective analysis of 1181 patients at MD Anderson Cancer Center, 94% of whom had received a median of 4 previous lines of therapy demonstrated a median survival of 10 months^18^. In addition, up to 20% of patients died within the first 90 days. These early deaths on study are most often attributable to disease progression, since most modern phase 1 studies are associated with a lower treatment related mortality of around 0.5% ^1,19,20^. Compared with other studies ^15,21–27^, we observed a 90 DM rate of 9.3%, with no treatment-related deaths. Majority of our patient population was heavily pre-treated (56% received >2 lines of treatment). This again underscores the impact of disease progression on early death on study. Another finding in our analysis is that patients with prolonged SD > 6 months had a similar OS as patients achieveing a CR and/or PR. This supports the observation that phase 1 trials are often associated with prolonged disease control despite low clinical anti-tumor response rates. In recent era, even in the absence of CR, an improved OS was demonstrated with PR or long term SD alone^16^. It was also shown that disease control (combined CR, PR and SD) has been associated with increased survival in patients on phase 1 trials ^16,26^.

The median overall survival in our cohort of 26.6 months is longer than that previously published reports by several other Phase 1 programs. (5.7–9.0 months)^16,22,26,28^. The clinical benefit rate in our cohort of 66% is comparable with that reported by other recent studies (45–56%)^16,21,29^. Moreover, the median time to treatment failure for our cohort where nearly two thirds of enrolled patients had received ≥ 3 prior treatments is comparable to the progression-free survival rates seen in recent FDA approved third or fourth-line therapies^30,31^. Patients stopped the study medication upon progression on corresponding clinical trial. Post – progression 48% of our cohort was treated on another clinical trial at our institution or elsewhere and 20 percent returned to primary oncologist to continue standard of care as SOC and the rest was managed by palliative care or hospice. Our data underscores the need to consider phase 1 clinical trials earlier rather than later especially in the setting where third and fourth line therapies provide marginal benefit and are associated with substantive toxicity^30^. As with any clinical trial, clinicians have to engage patients in a full discussion of the nature of the phase 1 trials and associated toxicity, risks and benefits.

Our study also reported higher than expected probability of an objective tumor response and survival among patients enrolled in phase 1 trials. It should be noted that there were no cytotoxic studies involved in our cohort compared to 4–16% in other reported series^16,28^. This is likely contributing to the improved outcomes compared to other published series.

One third of the patients in this cohort underwent next generation sequencing (NGS) through various platforms such as foundation one, strata, tempus or in-house pathology and half of them revealed actionable mutations. Only seven of our patients were enrolled on the single trial that required a specific alteration identified on NGS. Others had alterations including FGFR, BRCA1, MET, ATM, PD1, MGMT, BRAF, ALK, EGFR, TCR, BCL2. In the past two years, with tissue agnostic FDA drug indications in oncology, and development of biomarker driven clinical studies, the use of molecular profiles has increased significantly. Studies indicate that 30% of patients who undergo tumor genomic profiling may have an actionable alteration, which implies that patients may be matched to either an approved or investigational therapy^32^. Patients who received targeted agents matched to their oncogenic alterations had significantly better response rates of 27% compared to 5% in non-matched. This also translated to improved survival of 13 months in matched vs 9 months in unmatched patients^33^.

Although phase 1clinical trials are critical in the development of new cancer treatments, their progress is often limited by low accrual rates. Previously the barriers to the enrollment into phase I oncology trials included inability to meet all eligibility criteria, lack of treatment slots in the trials, and patient refusal^34^. At our center, over 90% of eligible patients consented to participation on a clinical trial and patient refusal has not been found to be a barrier. Ineligibility for enrollment due to poor performance status at the time of screening, lack of availability of trial slots and too many prior treatments have been the main reasons for ineligibility. Nearly half of our patient had received ≥3 lines of treatment (≥5 in 24%). The participation of African American patients in our Phase 1 trials of 20% approximates the African American population in the state of Alabama and race was not a barrier to participation in early phase trials. Lack of physician advocacy for a clinical trial has often been found to be a key cause for reduced participation of minorities on clinical trials^35^. The referral pattern indicated that majority of the patients were referred from in-house (71%) oncologists in the university compared to physician referrals from outside the university (29%). About one quarter of patients referred to the phase 1 Program did not meet the necessary eligibility criteria at screening. This is comparable to other retrospective reviews^36^.

The generalization of our findings is limited by the retrospective, single center nature of our study, the exclusively receipt of TT or IOT, and small number of included studies (17) with trial specific eligibility criteria enrolling diverse malignancies. Although heterogeneity of patients could be a confounder in our analysis, it was noted previously that tumor histology was not significant in predicting early mortality in patients treated in first-in- human studies ^19,22^. Our 90DM and OS should be viewed as exploratory in view of the <10% event rate contributing to 90DM.

During the last 20 years, the advent of targeted therapies in Phase 1 trials has improved clinical benefit in terms of overall survival rates and toxicity profiles when compared with the era of cytotoxic agents. Indeed, it is an exciting time for oncology discipline and Phase 1 clinical trials should not be viewed as a last resort for patients who have failed current therapy. Rather, enrollment in clinical trials should be viewed as another therapeutic option. As knowledge in the field of oncology continues to evolve, more precise targeting of molecular pathways will be expected to be unfolded.

## Conclusion

Modern first-in-human clinical trials represent a viable therapeutic option for many patients who progress through standard of care therapy. Phase1 trials have acceptable toxicity and are associated with overall improved outcomes. We conclude that phase I clinical trials should be considered for advanced cancer patients relatively early in the treatment phase, preferably upon progression of front line definitive systemic treatment.

## Data Availability

Data supporting the results reported in the article can be made available up on request.
